# Percutaneous closure of patent ductus arteriosus via internal jugular vein in patient with interrupted inferior vena cava

**DOI:** 10.4103/0974-2069.58321

**Published:** 2009

**Authors:** Nehal H Patel, Tarun H Madan, Amar M Panchal, Bhavesh M Thakkar

**Affiliations:** Department of Cardiology, U N Mehta Institute of Cardiology & Research Centre, Ahmedabad, India

**Keywords:** Patent ductus arteriosus, percutaneous closure, transjugular approach

## Abstract

Transcatheter closure of patent ductus arteriosus (PDA) using various occluders and coils via femoral vein is a well established therapeutic option. However, in patients with interrupted inferior vena cava (IVC) it is not feasible to close the PDA percutaneously using traditional methods. We present a nine-year-old girl with IVC interruption in whom percutaneous closure of PDA was successfully accomplished via the transjugular approach.

## CASE REPORT

A nine year old girl weighing 21 kg presented with a history of recurrent respiratory tract infections and failure to thrive. On clinical examination, she had a left ventricular (LV) apex, normal heart sounds and a continuous murmur in the left infraclavicular area. Her 12 lead Electrocardiogram (ECG) revealed sinus tachycardia with left atrial (LA) and LV enlargement. Chest X-ray showed cardiothoracic ratio of 60%, prominent pulmonary conus and plethoric lung fields. Transthoracic 2D echocardiography confirmed large conical PDA measuring 5.1 mm at its narrowest point with evidence of LA and LV volume overload. A spectral Doppler revealed continuous flow across the PDA with peak systolic gradient of 70 mmHg and end diastolic gradient of 38 mmHg. The right ventricular (RV) systolic pressure measured by tricuspid regurgitation (TR) velocity was 46 mmHg. With this data, it was decided to offer percutaneous closure of ductus to the patient.

### Procedure

**Figure 1:**
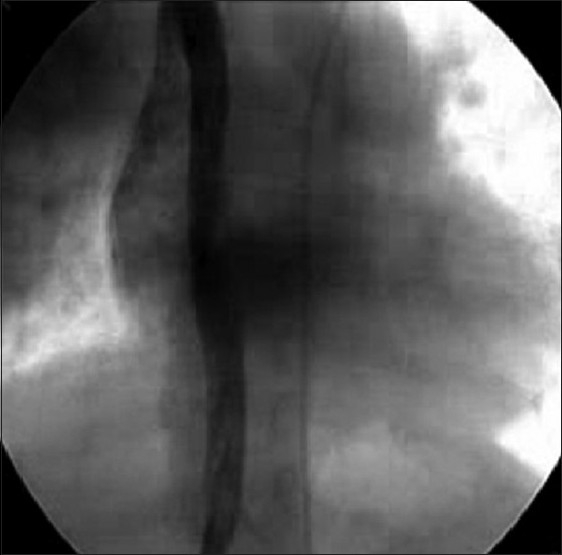
Fluoroscopic image in anteroposterior view showing interrupted inferior vena cava

**Figure 2:**
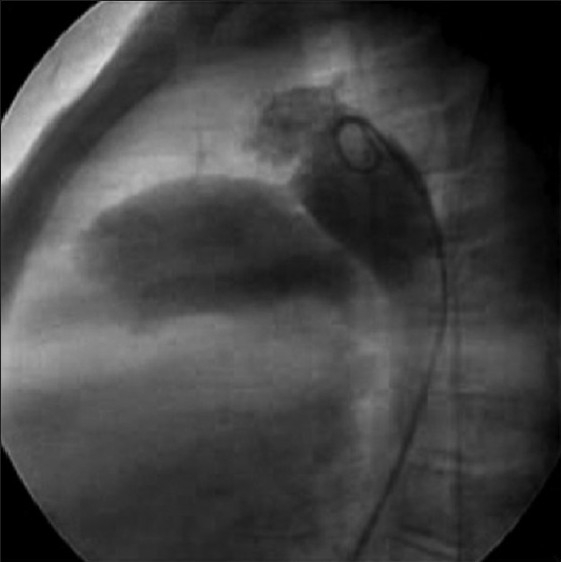
Fluoroscopic image of descending aortogram in lateral view showing moderate size PDA

Right femoral vein and artery were cannulated with 6F and 5F sheaths respectively. During the right heart catheterization, course of the catheter suggested IVC interruption with azygous continuation into right superior vena cava (SVC), which was confirmed with hand injection of contrast in the infrarenal portion of the IVC [Figure 1]. Hemodynamic data revealed systemic arterial pressure of 130/73/99 mmHg and pulmonary artery (PA) pressure of 68/41/53 mmHg. Descending aortogram in lateral view revealed moderate size conical PDA [Figure 2] with minimal diameter of 6.7 mm at the PA end. The aortic ampulla measured 12 mm.

**Figure 3:**
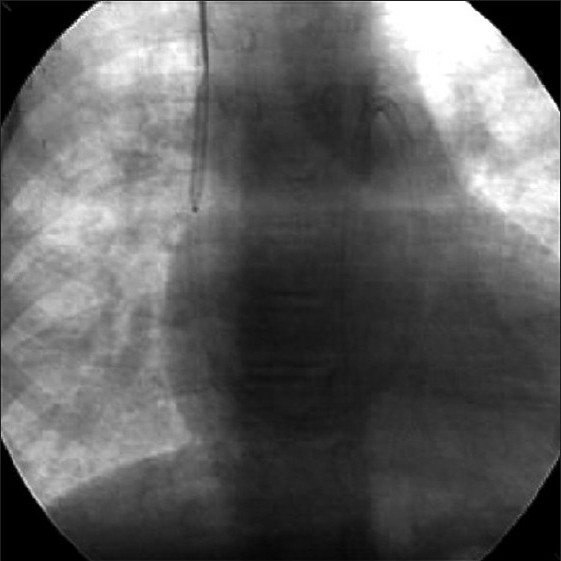
Fluoroscopic image of AP view showing multiple angulations of the delivery sheath with duct occluder

**Figure 4:**
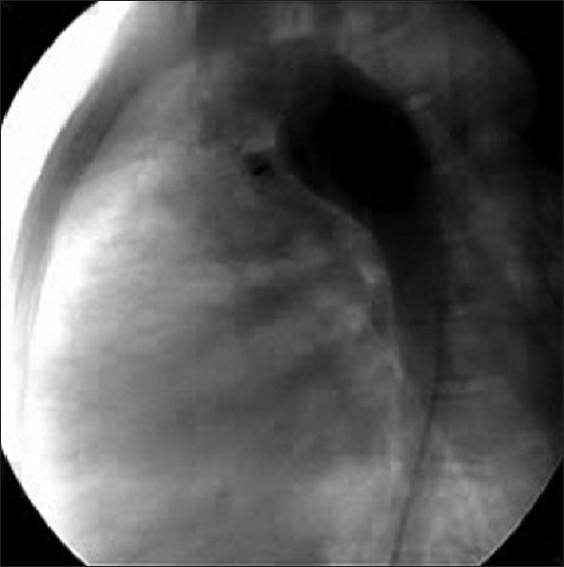
Fluoroscopic image of descending aortogram in lateral view showing PDA device *in situ* with minimal residual flow across it

After obtaining informed consent from the parents, it was decided to close the PDA through the right internal jugular vein (IJV), which was accessed with 6F sheath. A 6F Judkin’s right coronary catheter was advanced over J tip 0.035“ guide wire (Cordis Corporation, Miami, FL) from right IJV to main PA via the RV. Through this catheter 0.035” straight tip wire (Cordis Corporation, Miami, FL) was passed across the PDA into descending aorta. Considering the angulations in the catheter course, we preferred to exchange the 0.035“ wire with extra stiff Amplatz wire (AGA Medical Corp, Golden Valley, MN) for better support to position the delivery system across the PDA. An 8F delivery sheath was advanced and positioned in descending aorta over the Amplatz wire. Thus the sheath had to take an almost 90 degree turn from right atrium (RA) to PA and another 90 degree turn from PA to aorta. We did not encounter any difficulty while positioning the sheath or advancing the device through the sheath. Amplatz duct occluder (AGA Medical Corp, Golden Valley, MN) 12/10 mm size was screwed to the delivery cable and advanced through the delivery sheath [Figure 3] using the loader. The device was delivered using the standard technique. Lateral angiogram in the descending aorta revealed proper positioning of the device with mild residual flow through the device (“foaming”) [Figure 4]. Follow-up echocardiogram after 24 hours revealed trivial residual flow through the device. Doppler echocardiogram revealed TR velocity of ced to city reduced to 2.4 mmHg low revealed continuous flow with 2.4 m/sec with normal signals in the left PA and across the isthmus into the descending aorta.

## DISCUSSION

Clinical presentation of patients with PDA is variable. It predominantly depends upon the size of the PDA and the relationship between the systemic and pulmonary artery pressures and vascular resistances. Irrespective of the symptomatic status, clinically audible PDAs are closed surgically or through videoscopic assisted minimally invasive technique or percutaneously using coils or devices.[1‐6] With the advent of Amplatz duct occluder, almost all the PDAs beyond neonatal period and early infancy are closed with the transcatheter technique.[7]

Owing to large vessel diameter and easy vascular access, percutaneous interventions in pediatric patients are conventionally done via femoral vein and artery. However, in patients with interrupted IVC, as was the case in our patient, it is extremely difficult to deliver a device through femoral venous route. In such cases, alternative approach such as IJV or subclavian venous access needs to be considered. We preferred the IJV approach over the subclavian vein as the former has more direct access to the right side of the heart while the latter has a right angle entry into the right SVC. This bend could cause difficulty in manipulating the delivery sheath into the descending aorta across the PDA. There are a few case reports of PDA being closed percutaneously via IJV access.[8] Though the technique of transcatheter treatment of CHD via IJV is similar to the procedure via femoral vein, it is technically a little more challenging. Use of superstiff wire helped us track the sheath into the descending aorta across all the curves. A breaded sheath was used in this case to prevent kinking in the region of the right ventricular inflow and outflow, as well as in the PDA, and since it tracks down esily into the descending aorta. Another strategy would be to close the PDA from the arterial side using either Amplatzer duct occluder II or a slightly oversized Amplatzer duct occluder I or a muscular ventricular septal defect device.

PDA is not commonly associated with IVC interruption.[9] Though echocardiographic assessment of systemic venous drainage is essential prior to any percutaneous intervention, cardiac catheterization is the most reliable method to define the systemic drainage. While IVC interruption can come as a surprise during therapeutic catheterization, one should be aware of and prepared with alternative strategies.
